# Association between endometriosis and type and age of menopause: a pooled analysis of 279 948 women from five cohort studies

**DOI:** 10.1093/humrep/deaf068

**Published:** 2025-04-30

**Authors:** Hsin-Fang Chung, Kunihiko Hayashi, Annette J Dobson, Sven Sandin, Yuki Ideno, Rebecca Hardy, Elisabete Weiderpass, Gita D Mishra

**Affiliations:** Australian Women and Girls’ Health Research Centre, School of Public Health, The University of Queensland, Queensland, Australia; Gunma University Initiative for Advanced Research, Gunma University, Gunma, Japan; Gunma University Initiative for Advanced Research, Gunma University, Gunma, Japan; School of Health Sciences, Gunma University, Gunma, Japan; Australian Women and Girls’ Health Research Centre, School of Public Health, The University of Queensland, Queensland, Australia; Department of Medical Epidemiology and Biostatistics, Karolinska Institutet, Stockholm, Sweden; Department of Psychiatry, Icahn School of Medicine at Mount Sinai, New York, NY, USA; Gunma University Initiative for Advanced Research, Gunma University, Gunma, Japan; Centre for Food Science and Wellness, Gunma University, Gunma, Japan; School of Sport, Exercise and Health Sciences, Loughborough University, Loughborough, UK; International Agency for Research on Cancer, World Health Organisation, Lyon, France; Australian Women and Girls’ Health Research Centre, School of Public Health, The University of Queensland, Queensland, Australia; Gunma University Initiative for Advanced Research, Gunma University, Gunma, Japan

**Keywords:** endometriosis, surgical menopause, natural menopause, premature ovarian insufficiency, early menopause

## Abstract

**STUDY QUESTION:**

What is the association between endometriosis and the type and age of menopause?

**SUMMARY ANSWER:**

Women with endometriosis had a 7-fold increased risk of undergoing surgical menopause rather than natural menopause and were more likely to experience premature or early menopause, both surgically and naturally.

**WHAT IS KNOWN ALREADY:**

Endometriosis is associated with reduced ovarian reserve, but evidence on its relationship with the type of menopause (surgical vs natural) and timing (especially premature and early menopause) is limited. Women with endometriosis are more likely to undergo hysterectomy and/or oophorectomy (either unilateral or bilateral), but the average age of these surgeries remains unclear.

**STUDY DESIGN, SIZE, DURATION:**

The study analysed individual-level data from 279 948 women in five cohort studies conducted in the UK, Australia, Sweden, and Japan between 1996 and 2022.

**PARTICIPANTS/MATERIALS, SETTING, METHODS:**

Women whose menopause type and age could not be determined due to premenopausal hysterectomy with ovarian preservation or use of menopausal hormone therapy were excluded. Endometriosis was identified through self-reports and administrative data. Surgical menopause was defined as premenopausal bilateral oophorectomy. Fine–Gray subdistribution hazard models estimated hazard ratios (HRs) for surgical and natural menopause. Age at menopause was determined by the ages at the final menstrual period or bilateral oophorectomy. Linear regression assessed mean differences in menopause age, while multinomial logistic regression estimated odds ratios (ORs) for categorical menopause age: <40 (premature), 40–44 (early), 45–49, 50–51 (reference), 52–54, and ≥55 years. Spontaneous premature ovarian insufficiency (POI) was defined as natural menopause before age 40 years.

**MAIN RESULTS AND THE ROLE OF CHANCE:**

Endometriosis was identified in 3.7% of women. By the end of follow-up, 7.9% had surgical menopause and 58.2% experienced natural menopause. Using a competing risk model, women with endometriosis had a 7-fold increased risk of surgical menopause (HR: 7.54, 95% CI 6.84, 8.32) and were less likely to experience natural menopause (HR: 0.40, 95% CI 0.33, 0.49). On average, surgical menopause occurred 1.6 years (19 months) earlier (β: −1.59, 95% CI −1.77, −1.42) in women with endometriosis. Among women who experienced natural menopause, it was 0.4 years (5 months) earlier (β: −0.37, 95% CI −0.46, −0.28) for those with endometriosis. Women with endometriosis were twice as likely to experience premature surgical menopause (<40 years) (OR: 2.11, 95% CI 2.02, 2.20) or 1.4 times more likely to develop spontaneous POI (OR: 1.36, 95% CI 1.17, 1.59). They were also at increased odds of early surgical and natural menopause (40–44 years).

**LIMITATIONS, REASONS FOR CAUTION:**

This study could not differentiate between subtypes and stages of endometriosis or assess treatments for ovarian endometrioma, which may impact ovarian reserve. Self-reported menopause type and age could introduce recall bias.

**WIDER IMPLICATIONS OF THE FINDINGS:**

Given the consistent findings across individual studies, our results are likely to be generalizable to different populations, highlighting the need for tailored management of endometriosis to prevent medically induced or premature menopause. Long-term monitoring of women with endometriosis is recommended, given their elevated risk of surgical menopause and premature or early menopause, which are associated with adverse health outcomes in later life.

**STUDY FUNDING/COMPETING INTEREST(S):**

The InterLACE Consortium is funded by the Australian National Health and Medical Research Council project grant (APP1027196) and Centres of Research Excellence (APP1153420). G.D.M. is funded by the Australian National Health and Medical Research Council Leadership Fellowship (APP2009577). This research is funded in part by the Japan Society for the Promotion of Science (JSPS KAKENHI: 19KK0235, 23KK0167). The authors have no conflict of interest. Where authors are identified as personnel of the International Agency for Research on Cancer or WHO, the authors alone are responsible for the views expressed in this article, and they do not necessarily represent the decisions, policy, or views of the International Agency for Research on Cancer or WHO.

**TRIAL REGISTRATION NUMBER:**

N/A.

## Introduction

Endometriosis is a chronic, oestrogen-dependent inflammatory condition characterized by the presence of endometrium-like tissue outside the uterus (Zondervan *et al.*, 2020). The condition is estimated to affect 10% of women of reproductive age, though its prevalence varies considerably depending on population characteristics, diagnostic methods, and data sources (Shafrir *et al.*, 2018; Zondervan *et al.*, 2020). A meta-analysis estimated the prevalence of endometriosis to range from 1% to 5% in the general population (Sarria-Santamera *et al.*, 2020), with substantially higher rates (up to 50%) among women with infertility (Shafrir *et al.*, 2018). In Australia, the cumulative prevalence of clinically confirmed endometriosis was 6.0% by age 40–44 years (Rowlands *et al.*, 2021).

Endometriosis and its surgical treatment may damage ovarian reserve, as evidenced by lower levels of anti-Müllerian hormone (AMH) and antral follicle count, both of which can contribute to infertility (Macer and Taylor, 2012; Seyhan *et al.*, 2015; Muzii *et al.*, 2018; Tian *et al.*, 2021). However, limited evidence exists regarding its impact on the type and timing of menopause. Studies have reported that women with endometriosis are more likely to undergo hysterectomy and/or oophorectomy (either unilateral or bilateral) (Pokoradi *et al.*, 2011; Ainsworth *et al.*, 2022), but the average age at which these surgeries occur remains unclear. This is particularly relevant for premenopausal bilateral oophorectomy, which induces surgical menopause and is linked to an increased risk of cardiovascular disease (CVD) and premature death (Parker *et al.*, 2009a; Zhu *et al.*, 2020), especially when performed before age 45 years (Evans *et al.*, 2016; Zhu *et al.*, 2020).

Shared biological pathways (e.g. inflammation and autoimmune disorders) and common risk factors (e.g. early menarche, nulliparity, infertility, and low body weight) are believed to contribute to the association between endometriosis and earlier onset of natural menopause (Bertone-Johnson *et al.*, 2019; Mishra *et al.*, 2019; Zondervan *et al.*, 2020; Liang *et al.*, 2023). Two studies have suggested that women with endometriosis, particularly those with infertility attributed to endometriosis, experienced menopause early (before the median age) (Pokoradi *et al.*, 2011; Yasui *et al.*, 2011). To date, only one cohort study has examined the risk of early natural menopause, which is clinically defined as menopause before age 45 years in White women (Kulkarni *et al.*, 2022). A small clinical study of endometriosis patients (n = 302) investigated the development of premature ovarian insufficiency (POI, also known as premature menopause), defined as the loss of ovarian activities before age 40 years (Coccia *et al.*, 2011). This study reported a mean age at menopause of 45.3 ± 4.3 years, with 16% of patients experiencing POI (Coccia *et al.*, 2011), which was significantly higher than the 1–3% observed in the general population (Mishra *et al.*, 2019). Larger, more representative studies are needed to better quantify the age at menopause and the risk of POI among women with endometriosis.

This study aimed to examine the association between endometriosis and menopause type (surgical vs natural) as well as age at menopause, analysed both as a continuous and categorical variable, with a particular focus on premature and early menopause. We used individual-level data from five cohort studies participating in the International Collaboration for a Life Course Approach to Reproductive Health and Chronic Disease Events (InterLACE).

## Materials and methods

### Study participants

InterLACE is an ongoing international women’s health consortium that combines individual-level data from observational studies. A detailed description of the study design and data harmonization process has been previously published (Mishra *et al.*, 2016). This study combined data from five cohorts that collected information on endometriosis and menopause between 1996 and 2022: the Australian Longitudinal Study on Women’s Health (ALSWH) 1946-51 cohort, Women’s Lifestyle and Health Study (WLHS), Japan Nurses’ Health Study (JNHS), MRC National Survey of Health and Development (NSHD, known as the 1946 British Birth Cohort), and UK Biobank (n = 388 749).

We excluded women who had undergone premenopausal hysterectomy with ovarian preservation (n = 40 752; 10.5%), as their menopause age could not be determined, and those whose menopause status was unknown due to hormone use or insufficient data (n = 44 325; 11.4%). The sample included 303 672 women with data on menopause type (surgical, natural, or pre-/perimenopause). Additional exclusions were made for missing data on endometriosis (n = 3187; 0.8%), age at menopause (n = 1222; 0.3%), sociodemographics (n = 9764; 2.5%), or reproductive factors (n = 9551; 2.5%). The final analytic sample included 279 948 women with complete data. Women excluded due to missing data were more likely to be non-White, less educated, current smokers, and infertile compared with those included (Supplementary Table S1).

### Ethical approval

Ethical approval was obtained from the Institutional Review Board or Human Research Ethics Committee at each participating institution, and all participants provided informed consent. InterLACE used non-identifiable data from existing studies and received an ethics exemption from the University of Queensland (2024/HE000390).

### Exposure and outcome variables

Endometriosis diagnosis was determined through self-reported questionnaires across all studies and linked administrative data for the ALSWH, WLHS, and UK Biobank. Women were asked whether they had physician-diagnosed endometriosis. Additionally, the WLHS and JNHS collected information on infertility due to endometriosis, while the NSHD asked whether a hysterectomy was performed due to endometriosis (only including cases reporting hysterectomy with bilateral oophorectomy here). Administrative data from ALSWH, WLHS, and UK Biobank included hospital admissions, emergency records, or patient registries, where endometriosis was identified using ICD-10 code N80 or ICD-9 code 617. UK Biobank also included primary care data for endometriosis. A sub-cohort validation study of JNHS indicated that 87% of self-reported endometriosis cases were confirmed, with endometriosis sites verified in 94% of cases, supporting the reliability of self-reported data (Yasui *et al.*, 2011). Similarly, ALSWH data showed that 75% of endometriosis cases were identified through surveys, with half of these further validated by administrative records (Rowlands *et al.*, 2021).

The primary outcomes were the type and age of menopause. Natural menopause was defined as amenorrhea for at least 12 consecutive months, not attributable to hysterectomy, oophorectomy (unilateral or bilateral), or chemo-/radiotherapy. Surgical menopause was defined as the removal of both ovaries before menopause (premenopausal bilateral oophorectomy). Women who continued to have menstrual periods (regular or irregular) at the end of follow-up were classified as pre-/perimenopausal. Age at menopause was determined by self-reported age at the final menstrual period (natural menopause) or age at bilateral oophorectomy (surgical menopause). This information was collected retrospectively or prospectively across multiple surveys and analysed both as a continuous variable and a categorical variable. Based on previous literature (Zhu *et al.*, 2019; Chung *et al.*, 2023), menopause age was categorized as <40 (premature menopause), 40–44 (early menopause), 45–49, 50–51, 52–54, and ≥55 years, with the average menopause age of 50–51 years serving as the reference category. Spontaneous POI was defined as natural menopause before age 40 years.

### Covariates

Based on previous literature (Kulkarni *et al.*, 2022), several sociodemographic, lifestyle, and reproductive factors, collected at cohort entry or mid-age in the birth cohort (defined as baseline), were considered potential confounders and harmonized across studies. These factors included birth year (before 1940, 1940–1949, 1950–1959, or in 1960 or later), race (White, Black, Asian, or Mixed/Other), education level (no formal education, ≤10 years, 11–12 years, or university degree), smoking status (never, former, or current smoker), and BMI. Standard BMI cut-offs of <18.5, 18.5–24.9, 25–29.9, and ≥30 kg/m^2^ were used to categorize underweight, normal weight, overweight, and obese, respectively, for White, Black, and Mixed/Other women. Lower BMI cut-offs of <18.5, 18.5–22.9, 23–27.4, and ≥27.5 kg/m^2^ were used for Asian women (WHO Expert Consultation, 2004).

**Figure 1.**
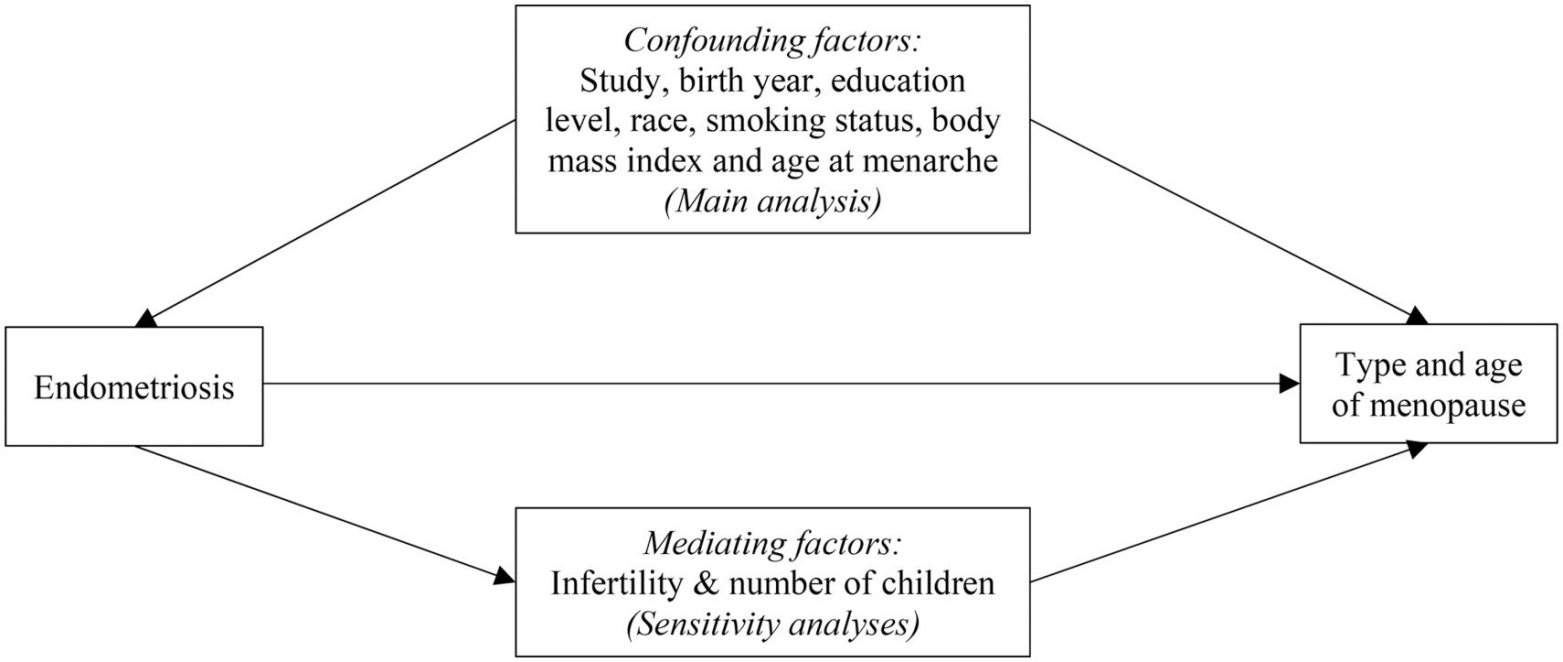
**Directed acyclic graph for the association between endometriosis and type and age of menopause, and their confounding and mediating factors**.

Key reproductive factors included age at menarche (≤11, 12, 13, 14, or ≥15 years), number of children (0, 1, 2, 3, or ≥4), and a history of infertility (yes or no) (Mishra *et al.*, 2019; Zondervan *et al.*, 2020; Liang *et al.*, 2023). Infertility and number of children were considered as potential mediators, because endometriosis is a common cause of infertility (Zondervan *et al.*, 2020), which can influence the number of children. Both infertility and nulliparity are established risk factors for premature or early menopause (Mishra *et al.*, 2019; Liang *et al.*, 2023). A directed acyclic graph illustrating these associations is presented in Fig. 1.

### Statistical analyses

Data from all cohorts were combined, and individual-level data were analysed. Participants contributed person-years from birth until the onset of surgical or natural menopause, 60 years of age, loss to follow-up, or the end of follow-up, whichever occurred first. We used Fine–Gray subdistribution hazard models to estimate hazard ratios (HRs) for surgical and natural menopause accounting for competing risks. When surgical menopause was the event of interest, natural menopause was treated as a competing event, and pre-/perimenopausal women were censored at their last reported menopausal status. Conversely, when natural menopause was the event of interest, surgical menopause was treated as a competing event. The proportional hazards assumption was assessed by Schoenfeld residuals and was found to be violated for birth year and education level. Study variability and within-study correlations were accounted for by including the study as a covariate and using robust variance estimators (crude model). Models were stratified by birth year and education level (as stratum variables) and adjusted for categorical covariates, including race, smoking status, and BMI (model 1), as well as age at menarche (model 2).

Generalized estimating equation (GEE) models were used to examine the association between endometriosis and age at menopause in postmenopausal women (n = 185 002) while accounting for correlated data. Study variability and within-study correlations were accounted for by including study as a covariate and indicating study as a cluster. We fitted separate models for natural and surgical menopause. Menopause age was initially treated as a continuous variable, and linear regression models were used to estimate mean differences in menopause age between women with and without endometriosis, while adjusting for the same covariates as described above. We used multinomial logistic regression models to estimate odds ratios (ORs) for categorical menopause age, with menopause at 50–51 years serving as the reference category.

We conducted several secondary and sensitivity analyses. First, to explore whether the associations were partially mediated by infertility and number of children, these factors were included as additional covariates. Second, studies relying solely on self-reported survey data for endometriosis were excluded, as they tended to report higher prevalence rates than other studies. Third, study-level analyses were performed to assess between-study heterogeneity and determine if any single study disproportionately influenced the overall estimates. Because of the smaller sample size in individual studies, we combined the <40 and 40–44 years age groups into a single early menopause category (<45 years). A random-effects meta-analysis, known as the two-step method, was used to examine heterogeneity across study-level estimates, assessed using *I*^2^ statistics and *P*-values. Regression models were fitted using the PHREG and GEE procedures in SAS 9.4, while the METAN procedure in STATA 17.0 was used for meta-analysis.

## Results

### Study characteristics

The study included 279 948 women from five cohort studies (Table 1), of whom 10 367 (3.7%) reported endometriosis, with over half of these cases identified through administrative data. The prevalence of endometriosis ranged from 2.2% in the WLHS (ascertained from patient registries and a single survey) to 6.0% in the JNHS (six surveys). By the end of follow-up, 58.2% of women had experienced natural menopause (mean age: 50.5 ± 4.3 years), and 7.9% had undergone surgical menopause (mean age: 47.2 ± 6.6 years). When examining only postmenopausal women, the rate of surgical menopause ranged from 10% to 15% across studies in Western countries, while it was 4% in the JNHS (Japanese nurses). Table 2 shows the baseline characteristics based on endometriosis and menopause type. Women born in 1960 or later, of Asian descent, with early menarche (≤11 years), nulliparity, and infertility were more likely to have endometriosis. Women who were less educated, smokers, obese, and had early menarche were more likely to undergo surgical menopause.

**Table 1.** Characteristics of five cohort studies with information on endometriosis and menopause in the InterLACE consortium.

Study	Country	Sample	Endometriosis			Type of menopause^b^			Age at menopause	
			Data sources	Cases N (%)	Linked (%)^a^	Surgical N (%)	Natural N (%)	Pre-/peri N (%)	Surgical M ± SD	Natural M ± SD
Australian Longitudinal Study on Women’s Health (ALSWH)	Australia	7655	Hospital admissions, emergency and self-report (1 survey)	420 (5.5)	37.4	1104 (14.4)	6223 (81.3)	328 (4.3)	46.3 ± 6.9	50.9 ± 4.2
Women’s Lifestyle and Health Study (WLHS)	Sweden	27 880	Patient registries and self-report (1 survey)	620 (2.2)	43.9	1127 (4.0)	10 131 (36.3)	16 622 (59.6)	50.6 ± 6.2	50.7 ± 4.0
Japan Nurses’ Health Study (JNHS)	Japan	39 699	Self-report (6 surveys)	2384 (6.0)	0	372 (0.9)	8659 (21.8)	30 668 (77.3)	44.7 ± 6.7	50.1 ± 3.2
MRC National Survey of Health and Development (NSHD)	UK	638	Self-report (2 surveys)	29 (4.6)	0	79 (12.4)	550 (86.2)	9 (1.4)	46.9 ± 5.7	50.2 ± 3.3
UK Biobank (UK Biobank)	UK	204 076	Hospital admissions, primary care and self-report (1 survey)	6914 (3.4)	72.9	19 364 (9.5)	137 393 (67.3)	47 319 (23.2)	47.1 ± 6.6	50.5 ± 4.4
Total		279 948		10 367 (3.7)	52.8	22 046 (7.9)	162 956 (58.2)	94 946 (33.9)	47.2 ± 6.6	50.5 ± 4.3

Data were presented as number (%) or mean ± SD (M ± SD).

a Linked administrative data included hospital admissions, emergency, patient registries, or primary care data.

b Surgical menopause indicated bilateral oophorectomy before natural menopause.

**Table 2.** Baseline characteristics of participants according to history of endometriosis and type of menopause.

	History of endometriosis		Type of menopause		
Baseline characteristics	Yes (n = 10 367)	No (n = 269 581)	Surgical (n = 22 046)	Natural (n = 162 956)	Pre-/peri (n = 94 946)
Birth year					
Born before 1940	84 (0.8)	6170 (2.3)	726 (3.3)	5528 (3.4)	0 (0)
Born 1940–1949	2617 (25.2)	101 959 (37.8)	12 367 (56.1)	90 338 (55.4)	1871 (2.0)
Born 1950–1959	3865 (37.3)	95 293 (35.4)	7257 (32.9)	59 188 (36.3)	32 713 (34.5)
Born 1960 or later	3801 (36.7)	66 159 (24.5)	1696 (7.7)	7902 (4.9)	60 362 (63.6)
Race					
White	7613 (73.4)	222 348 (82.5)	20 811 (94.4)	148 894 (91.4)	60 256 (63.5)
Black	125 (1.2)	3500 (1.3)	363 (1.6)	1711 (1.0)	1551 (1.6)
Asian	2541 (24.5)	41 815 (15.5)	708 (3.2)	11 322 (6.9)	32 326 (34.1)
Mixed/Other	88 (0.9)	1918 (0.7)	164 (0.7)	1029 (0.6)	813 (0.9)
Education level					
No formal education	190 (1.8)	6796 (2.5)	669 (3.0)	4108 (2.5)	2209 (2.3)
≤10 years	3652 (35.2)	99 766 (37.0)	11 603 (52.6)	71 523 (43.9)	20 292 (21.4)
11–12 years	4057 (39.1)	89 059 (33.0)	4780 (21.7)	40 471 (24.8)	47 865 (50.4)
University degree	2468 (23.8)	73 960 (27.4)	4994 (22.7)	46 854 (28.8)	24 580 (25.9)
Smoking status at baseline					
Never smoker	6236 (60.2)	159 990 (59.4)	12 387 (56.2)	94 728 (58.1)	59 111 (62.3)
Former smoker	2796 (27.0)	79 114 (29.4)	7341 (33.3)	52 713 (32.4)	21 856 (23.0)
Current smoker	1335 (12.9)	30 477 (11.3)	2318 (10.5)	15 515 (9.5)	13 979 (14.7)
BMI at baseline^a^					
Underweight	279 (2.7)	5755 (2.1)	157 (0.7)	1835 (1.1)	4042 (4.3)
Normal weight	4645 (44.8)	124 345 (46.1)	7299 (33.1)	68 579 (42.1)	53 112 (55.9)
Overweight	3353 (32.3)	88 913 (33.0)	8200 (37.2)	59 044 (36.2)	25 022 (26.4)
Obese	2090 (20.2)	50 568 (18.8)	6390 (29.0)	33 498 (20.6)	12 770 (13.5)
Age at menarche					
≤11 years	2292 (22.1)	51 176 (19.0)	5152 (23.4)	30 529 (18.7)	17 787 (18.7)
12 years	2335 (22.5)	57 029 (21.2)	4355 (19.8)	32 169 (19.7)	22 840 (24.1)
13 years	2495 (24.1)	68 493 (25.4)	5044 (22.9)	41 101 (25.2)	24 843 (26.2)
14 years	1916 (18.5)	52 872 (19.6)	3991 (18.1)	33 030 (20.3)	17 767 (18.7)
≥15 years	1329 (12.8)	40 011 (14.8)	3504 (15.9)	26 127 (16.0)	11 709 (12.3)
Number of children					
0	2800 (27.0)	50 561 (18.8)	3748 (17.0)	26 508 (16.3)	23 105 (24.3)
1	1794 (17.3)	35 317 (13.1)	2887 (13.1)	20 584 (12.6)	13 640 (14.4)
2	3873 (37.4)	115 738 (42.9)	9794 (44.4)	73 354 (45.0)	36 463 (38.4)
3	1472 (14.2)	51 075 (19.0)	4059 (18.4)	31 583 (19.4)	16 905 (17.8)
≥4	428 (4.1)	16 890 (6.3)	1558 (7.1)	10 927 (6.7)	4833 (5.1)
History of infertility					
No	8806 (84.9)	256 393 (95.1)	21 197 (96.1)	157 654 (96.8)	86 348 (90.9)
Yes	1561 (15.1)	13 180 (4.9)	849 (3.9)	5294 (3.2)	8598 (9.1)

Data were presented as number (%).

a Standard BMI cut-offs <18.5, 18.5–24.9, 25–29.9, and ≥30 kg/m^2^ were used to categorize underweight, normal weight, overweight, and obese, respectively, in White, Black, and Mixed/Other women, while lower cut-offs <18.5, 18.5–22.9, 23–27.4, and ≥27.5 kg/m^2^ were used in Asian women (reference in the Materials and methods section).

### Endometriosis and type of menopause

The incidence of surgical menopause was significantly higher among women with endometriosis compared with those without endometriosis (7.3 vs 1.4 per 1000 person-years), while the incidence of natural menopause was lower (6.3 vs 12.3 per 1000 person-years) (Table 3). Using a competing risk model, women with endometriosis had a 7-fold increased risk of surgical menopause (HR: 7.54, 95% CI 6.84, 8.32) and were less likely to experience natural menopause (HR: 0.40, 95% CI 0.33, 0.49).

**Table 3.** The associations between history of endometriosis and type of menopause (n = 279 948).

	Sample		Surgical/natural cases	Incidence	**Crude model** ^a^	**Model 1** ^a^ ^,^ ^b^	**Model 2** ^a^ ^,^ ^c^
Type of menopause	N	Person-years	n (%)	/10^3^ p-yrs	HR (95% CI)	HR (95% CI)	HR (95% CI)
Surgical menopause							
Endometriosis							
Yes	10 367	477 673	3479 (33.6)	7.3	7.37 (6.92, 7.86)	7.58 (6.87, 8.36)	7.54 (6.84, 8.32)
No	269 581	12 970 097	18 567 (6.9)	1.4	Reference	Reference	Reference
Natural menopause							
Endometriosis							
Yes	10 367	477 673	3029 (29.2)	6.3	0.37 (0.27, 0.52)	0.40 (0.32, 0.49)	0.40 (0.33, 0.49)
No	269 581	12 970 097	159 927 (59.3)	12.3	Reference	Reference	Reference

Data were presented as number (%), incidence rate (per 1000 person-years), or hazard ratio (HR) and 95% CI.

a Fine–Gray subdistribution hazards models were used to examine the type of menopause and account for a competing risk. When surgical menopause was the event of interest, natural menopause was treated as a competing risk. In contrast, when natural menopause was the event of interest, surgical menopause was treated as a competing risk. Study variability and within-study correlation were accounted for by including an indicator for study as a covariate and using robust variance estimators (crude model).

b Model 1 was adjusted for study, race, smoking status, and BMI at baseline and stratified by birth year and education level (as stratum variables).

c Model 2 was adjusted for covariates in Model 1 and age at menarche.

### Endometriosis and age at menopause

Among women who experienced surgical menopause (n = 22 046), those with endometriosis had a younger mean age at menopause (45.2 ± 6.7 years) compared with those without endometriosis (47.6 ± 6.5 years) (Table 4). In linear regression models, endometriosis was associated with an earlier age at surgical menopause by 1.6 years (β: −1.59, 95% CI −1.77, −1.42). For categorical menopause age, a higher proportion of women with endometriosis experienced premature (<40 years) and early surgical menopause (40–44 years) compared with those without endometriosis (20.0% vs 11.8% and 24.1% vs 16.6%, respectively). In multinomial logistic regression models, endometriosis was strongly associated with premature (OR: 2.11, 95% CI 2.02, 2.20) and early surgical menopause (OR: 1.82, 95% CI 1.73, 1.92) compared with menopause at age 50–51 years.

**Table 4.** The associations between history of endometriosis and age at menopause among postmenopausal women (n = 185 002).

	History of endometriosis		**Crude model** ^a^	**Model 1** ^a^ ^,^ ^b^	**Model 2** ^a^ ^,^ ^c^
Age at menopause	Yes	No	β (95% CI) or OR (95% CI)	β (95% CI) or OR (95% CI)	β (95% CI) or OR (95% CI)
Surgical menopause (n = 22 046)					
Continuous age	45.2 ± 6.7	47.6 ± 6.5	−2.27 (−2.46, −2.08)	−1.59 (−1.78, −1.41)	−1.59 (−1.77, −1.42)
Categorical age					
<40 years	697 (20.0)	2186 (11.8)	2.41 (2.19, 2.64)	2.10 (2.02, 2.19)	2.11 (2.02, 2.20)
40–44 years	839 (24.1)	3073 (16.6)	2.12 (1.96, 2.29)	1.82 (1.73, 1.92)	1.82 (1.73, 1.92)
45–49 years	993 (28.5)	5745 (30.9)	1.32 (1.13, 1.54)	1.21 (1.06, 1.38)	1.21 (1.06, 1.38)
50–51 years	320 (9.2)	2450 (13.2)	Reference	Reference	Reference
52–54 years	320 (9.2)	2267 (12.2)	1.08 (1.03, 1.14)	1.10 (1.05, 1.15)	1.10 (1.05, 1.15)
≥55 years	310 (8.9)	2846 (15.3)	0.84 (0.73, 0.96)	0.98 (0.82, 1.16)	0.98 (0.82, 1.16)
Natural menopause (n = 162 956)					
Continuous age	49.9 ± 4.3	50.5 ± 4.3	−0.52 (−0.64, −0.40)	−0.38 (−0.46, −0.29)	−0.37 (−0.46, −0.28)
Categorical age					
<40 years	60 (2.0)	2512 (1.6)	1.41 (1.20, 1.65)	1.37 (1.17, 1.60)	1.36 (1.17, 1.59)
40–44 years	229 (7.6)	10 768 (6.7)	1.25 (1.14, 1.37)	1.25 (1.13, 1.38)	1.25 (1.13, 1.38)
45–49 years	844 (27.9)	37 337 (23.3)	1.19 (1.12, 1.27)	1.16 (1.09, 1.22)	1.15 (1.09, 1.22)
50–51 years	740 (24.3)	38 880 (24.3)	Reference	Reference	Reference
52–54 years	768 (25.4)	43 341 (27.1)	0.95 (0.89, 1.02)	0.96 (0.91, 1.02)	0.96 (0.91, 1.02)
≥55 years	388 (12.8)	27 089 (16.9)	0.86 (0.77, 0.91)	0.96 (0.87, 1.06)	0.96 (0.87, 1.06)

Data were presented as mean±SD, number (%), β and 95% CI, or odds ratio (OR) and 95% CI.

a Generalized estimating equation models were used to examine continuous and categorical age at menopause and account for correlated data. Study variability and within-study correlation were accounted by including an indicator for study as a covariate and indicating study as a cluster (crude model).

b Model 1 was adjusted for study, birth year, education level, race, smoking status, and BMI at baseline.

c Model 2 was adjusted for covariates in Model 1 and age at menarche.

Among women who experienced natural menopause (n = 162 956), those with endometriosis had a younger mean age at menopause (49.9 ± 4.3 years) compared with those without endometriosis (50.5 ± 4.3 years) (Table 4). After adjusting for covariates, endometriosis was associated with a 0.4 years earlier age (β: −0.37, 95% CI −0.46, −0.28) at natural menopause. For categorical age at natural menopause, endometriosis was associated with spontaneous POI (OR: 1.36, 95% CI 1.17, 1.59) and early menopause (OR: 1.25, 95% CI 1.13, 1.38).

### Secondary/sensitivity analyses

Additional adjustment for infertility and number of children slightly attenuated the observed associations, particularly the association between endometriosis and POI. The ORs for POI were attenuated from 1.36 (95% CI 1.17, 1.59) to 1.23 (95% CI 1.11, 1.36), suggesting that the association was partially mediated by infertility and nulliparity (Supplementary Table S2). Excluded studies that identified endometriosis solely through self-reported questionnaires did not alter the results (Supplementary Table S3). The two-step meta-analysis of individual studies yielded similar effect estimates. All studies demonstrated that endometriosis was associated with an increased risk of surgical menopause (pooled HR: 7.90, 95% CI 6.36, 9.44) and a decreased risk of natural menopause (pooled HR: 0.47, 95% CI 0.30, 0.64), although statistically significant between-study heterogeneity was observed (*I*^2^>89%, *P* < 0.001) (Supplementary Fig. S1). Consistent effect estimates were also found for early surgical menopause before age 45 years (pooled OR: 1.94, 95% CI 1.68, 2.21) and early natural menopause before age 45 years (pooled OR: 1.22, 95% CI 0.99, 1.45), with no statistically significant heterogeneity between studies (*P* > 0.1) (Supplementary Fig. S2).

## Discussion

The prevalence of endometriosis ranged from 2% to 6% across the studies, consistent with findings from previous meta-analyses (Sarria-Santamera *et al.*, 2020). Using a competing risk model, we found that women with endometriosis were seven times more likely to undergo surgical menopause and less likely to experience natural menopause. On average, they underwent surgical menopause 1.6 years (19 months) earlier, and for those who experienced natural menopause, it occurred 0.4 years (5 months) earlier. Women with endometriosis were twice as likely to have premature surgical menopause (<40 years) or 1.4 times more likely to develop premature natural menopause (spontaneous POI) compared with menopause at age 50–51 years. They were also more likely to have early surgical or natural menopause (40–44 years). These findings suggest that endometriosis is a risk factor for both surgical menopause and spontaneous POI or early menopause.

This study quantified the association between endometriosis and age at both natural and surgical menopause, an area not previously explored. A Japanese study reported that women with endometriosis experienced earlier natural menopause (48.8 years) compared with those without endometriosis (49.6 years), but it did not adjust for confounders using multivariable models or report data for women undergoing surgical menopause (Yasui *et al.*, 2011). Additionally, there is limited evidence of the association between endometriosis and POI or early menopause. Our findings align with those from the Nurses’ Health Study II, which found that laparoscopically confirmed endometriosis was associated with a 30% increased risk (HR: 1.28, 95% CI 1.10, 1.48) of early natural menopause <45 years (Kulkarni *et al.*, 2022). This effect estimate is similar to ours, where endometriosis was associated with a 30–40% increased odds of POI or early natural menopause. These estimates are likely smaller than those for surgical menopause, as surgery procedures tend to select women with endometriosis who are at a higher risk of early natural menopause. Only one study assessed the development of spontaneous POI, but all participants were clinical patients who had undergone laparoscopy for endometriosis (without a healthy control group) (Coccia *et al.*, 2011). Women who underwent bilateral endometrioma excision had a very young age at natural menopause (42.1 ± 5.1 years) and a high proportion of POI (36.4%), but no multivariable models were used to quantify the risk of POI (Coccia *et al.*, 2011). This pooled analysis of five studies, with a sufficiently large sample size, provides robust estimates of POI for both natural and surgical menopause.

Several plausible mechanisms have been proposed to explain the association between endometriosis and premature or early natural menopause, including reduced ovarian reserve, particularly in women with endometriomas, surgical excision, and advantaged stages (Seyhan *et al.*, 2015; Muzii *et al.*, 2018; Tian *et al.*, 2021). Women with endometriosis generally exhibit lower levels of AMH and antral follicle count and elevated levels of follicle-stimulating hormone compared with those without endometriosis or healthy controls (Seyhan *et al.*, 2015; Muzii *et al.*, 2018; Tian *et al.*, 2021). A meta-analysis showed that AMH levels decreased by up to 40% following ovarian cystectomy for endometriomas, and this decline appears to be permanent (Raffi *et al.*, 2012). Extraovarian endometriosis, while less impactful than endometrioma, can also have deleterious effects on the ovaries (Seyhan *et al.*, 2015). Moreover, endometriosis may impact fertility through mechanisms such as peritoneal inflammation, immune system dysfunction, pelvic adhesions, and endocrine derangements, all of which disrupt the follicular environment and reduce oocyte yield or ovarian reserve (Macer and Taylor, 2012). Our analysis suggests that the association between endometriosis and premature or early natural menopause may be partially mediated by infertility and nulliparity. There is also evidence suggesting an association between endometriosis and comorbid autoimmune disorders, such as thyroid disease, Addison’s disease, and systemic lupus erythematosus, which may contribute to an increased risk of POI or early menopause (European Society for Human Reproduction and Embryology (ESHRE) Guideline Group on POI *et al.*, 2016; Shigesi *et al.*, 2019).

Several hormone therapies (e.g. oral contraceptives, progestins) and conservative surgical options (e.g. lesion excision/ablation) are available to manage the symptoms of endometriosis before considering a hysterectomy (Zondervan *et al.*, 2020). In more severe cases, a hysterectomy with bilateral oophorectomy may be necessary, which can lead to early surgical menopause in premenopausal women. Bilateral oophorectomy at the time of hysterectomy for benign conditions has traditionally been performed to prevent ovarian cancer (Parker *et al.*, 2009b). A meta-analysis of 24 studies reported nearly a 2-fold increased risk of ovarian cancer associated with endometriosis (RR: 1.93, 95% CI 1.68, 2.22), with the risk primarily driven by the presence of endometrioma (Kvaskoff *et al.*, 2021). However, the study noted that ovarian cancer remains rare (1.3% in the general population) regardless of endometriosis status (Kvaskoff *et al.*, 2021). Based on this risk estimate, the absolute risk of ovarian cancer in women with endometrioma is approximately 2.5%, which is still relatively low. Therefore, interventions like bilateral oophorectomy to prevent ovarian cancer are not considered justified in current practices (Kvaskoff *et al.*, 2017). Despite this, our findings indicate that women with endometriosis are seven times (ranging 5–10 times across studies) more likely to undergo bilateral oophorectomy, especially before age 45 years. Further large-scale prospective studies are needed to investigate this association in more recent cohorts, as the prevalence of endometriosis has increased (Le Moal *et al.*, 2022; Australian Institute of Health and Welfare, 2023), and bilateral oophorectomy is not recommended for benign conditions.

### Strengths and limitations

This is the largest and most comprehensive study to date examining the type and timing of menopause among women with endometriosis. With a large, population-based sample, the study has sufficient statistical power to detect associations with rare outcomes, such as spontaneous POI. The use of competing risk methods allowed for the analysis of both the time to the first observed event and the type of the first event. Given the consistent findings across individual studies, our results are likely to be generalizable to different populations.

However, this study has several limitations. First, while over half of the endometriosis cases were identified through linked administrative data, we could not differentiate between subtypes and stages of endometriosis or determine whether women received surgical excision or ablation for ovarian endometrioma, which may impact ovarian reserve. It is important to note that women undergoing hysterectomy with ovarian preservation were excluded from the analysis, so hysterectomy would not explain the earlier natural menopause observed in this study. Second, self-reported age at menopause may introduce recall bias. However, previous studies indicate high reliability, with 72–82% of women recalling their menopause age within 1 year of prospectively recorded data (validity) or within 1 year between two follow-up surveys (reproducibility) (Colditz *et al.*, 1987; den Tonkelaar, 1997). The agreement was higher among women with surgical menopause. Although validity decreased over time, the mean difference between retrospectively and prospectively reported ages remained only 0.5 years, even 7–9 years after menopause (den Tonkelaar, 1997). Misclassification was less likely in cases of POI or early menopause, as these women would more readily notice an unexpectedly early cessation of menstruation. Third, although we adjusted for a wide range of covariates, including reproductive factors, the possibility of residual confounding remains. In particular, environmental factors such as exposure to endocrine-disrupting chemicals (Smarr *et al.*, 2016; Ding *et al.*, 2022) and variations in genetic predisposition across different cohorts may contribute to unmeasured confounding.

### Clinical implications

The current management of endometriosis primarily focuses on pain, infertility, and medical/surgical treatment following diagnosis (Johnson *et al.*, 2013; National Institute of Health and Care Excellence, 2017; Royal Australian and New Zealand College of Obstetricians and Gynaecologists, 2021; European Society of Human Reproduction and Embryology, 2022). While the ESHRE guidelines include menopause and menopause-related health concerns, the recommendations are mostly around the treatment of endometriosis in postmenopausal women (European Society of Human Reproduction and Embryology, 2022). Our robust evidence on the type and age of menopause may guide updates to endometriosis management guidelines, emphasizing prevention and tailored strategies for early or medically induced menopause. Long-term monitoring of women with endometriosis is recommended as they are at an elevated risk of surgical menopause and POI or early menopause, as these conditions are associated with adverse health outcomes, particularly CVD and premature death (Parker *et al.*, 2009a; Zhu *et al.*, 2019, 2020; Chung *et al.*, 2023). Modifying surgical approaches in the management of endometriosis could help reduce the incidence of premature or early surgical menopause.

Menopausal hormone therapy (MHT) is recommended for women experiencing POI until the average age of natural menopause as a primary prevention strategy to reduce morbidity and mortality (Panay *et al.*, 2024). However, in women with a history of endometriosis, MHT, particularly estrogen-only therapy, may have adverse effects, including the potential for disease recurrence and a small but possible risk of malignant transformation (Moen *et al.*, 2010; Gemmell *et al.*, 2017). Therefore, the most recent ESHRE guidelines recommend that women with POI and a history of endometriosis should receive combined estrogen–progestogen therapy, even in cases where a hysterectomy has been performed (Panay *et al.*, 2024).

## Conclusions

This pooled study provided robust evidence that women with endometriosis had a 7-fold increased risk of surgical menopause and were less likely to experience natural menopause. They tended to reach menopause earlier and were more likely to experience premature or early menopause, both surgically and naturally. Future studies incorporating data on the subtype, stage, and treatment of endometriosis are needed to better understand the biological mechanisms and evolutionary origins of the condition.

## Supplementary Material

deaf068_Supplementary_Figure_S1

deaf068_Supplementary_Figure_S2

deaf068_Supplementary_Table_S1

deaf068_Supplementary_Table_S2

deaf068_Supplementary_Table_S3

## Data Availability

The datasets that were generated for conducting this pooled analysis are not publicly available, but data from some individual studies can be accessed by submitting an application, for example, Australian Longitudinal Study on Women’s Health (https://alswh.org.au/for-data-users/applying-for-data/) and UK Biobank (https://www.ukbiobank.ac.uk/enable-your-research/apply-for-access).
