# Supplementary material for: Association between endometriosis and type and age of menopause: a pooled analysis of 279 948 women from five cohort studies
Source: Hum Reprod. 2025 Apr 30;40(6):1210–9. doi: 10.1093/humrep/deaf068 (PMC12127511; doi:10.1093/humrep/deaf068)
Supplement: deaf068_Supplementary_Figure_S1 [file deaf068_supplementary_figure_s1.pdf]

Human Reproduction, 2025, 1–1
https://doi.org/10.1093/humrep/deaf068
Supplementary Data

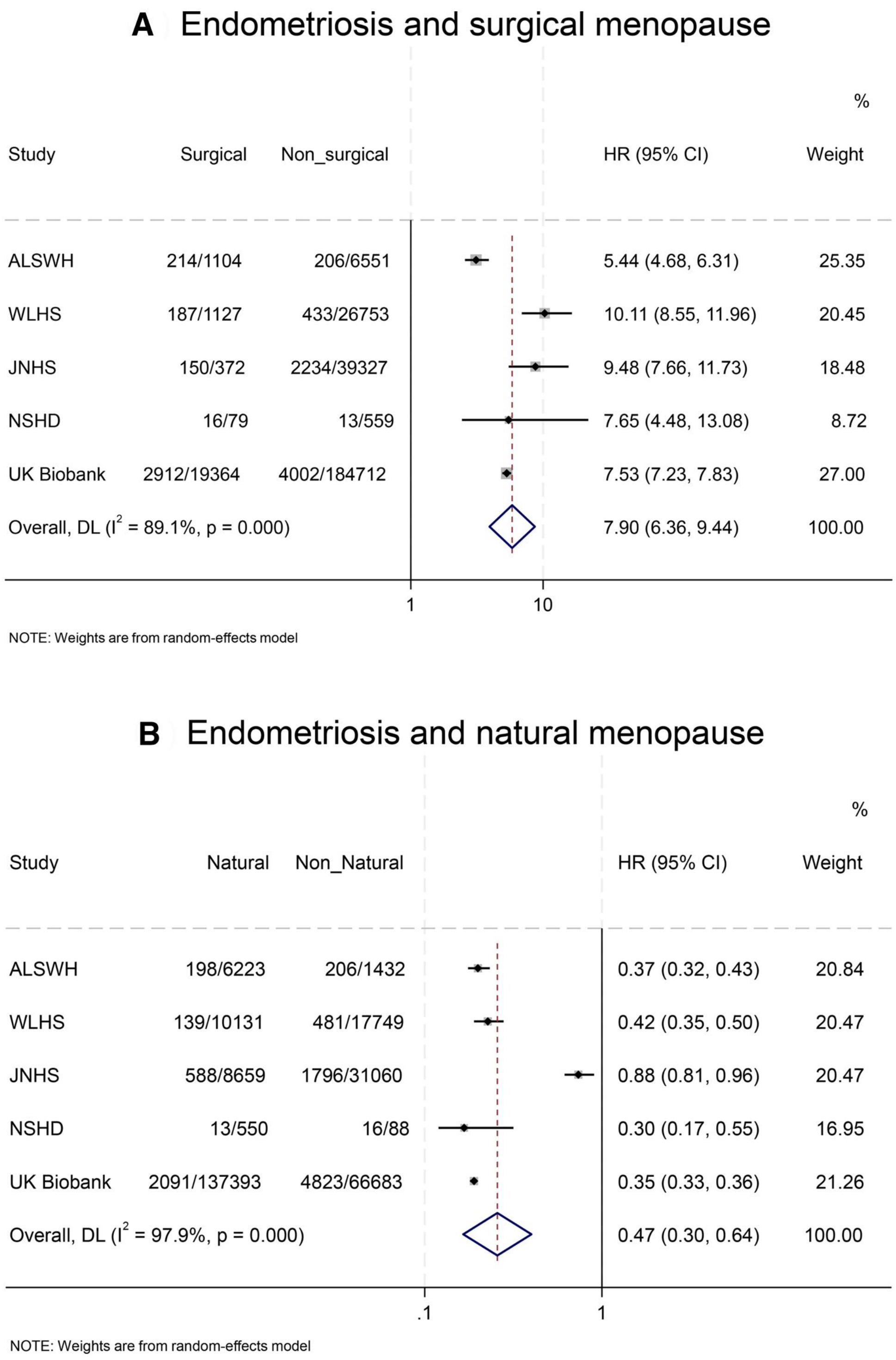

Supplementary Figure S1. Random-effect meta-analysis of the association between history of endometriosis and menopause. (**A**) Surgical menopause and (**B**) natural menopause. Fine–Gray subdistribution hazards models were used to account for competing risks, and hazard ratios (HRs) were fully adjusted for birth year, education level, race, smoking status, BMI, and age at menarche. DerSimonian–Laird (DL) method was used to estimate the heterogeneity variance (I^2 and *P*-values).
