# Supplementary material for: Association between endometriosis and type and age of menopause: a pooled analysis of 279 948 women from five cohort studies
Source: Hum Reprod. 2025 Apr 30;40(6):1210–9. doi: 10.1093/humrep/deaf068 (PMC12127511; doi:10.1093/humrep/deaf068)
Supplement: deaf068_Supplementary_Figure_S2 [file deaf068_supplementary_figure_s2.pdf]

OXFORD
human reproduction

Human Reproduction, 2025, 1–1
https://doi.org/10.1093/humrep/deaf068
Supplementary Data

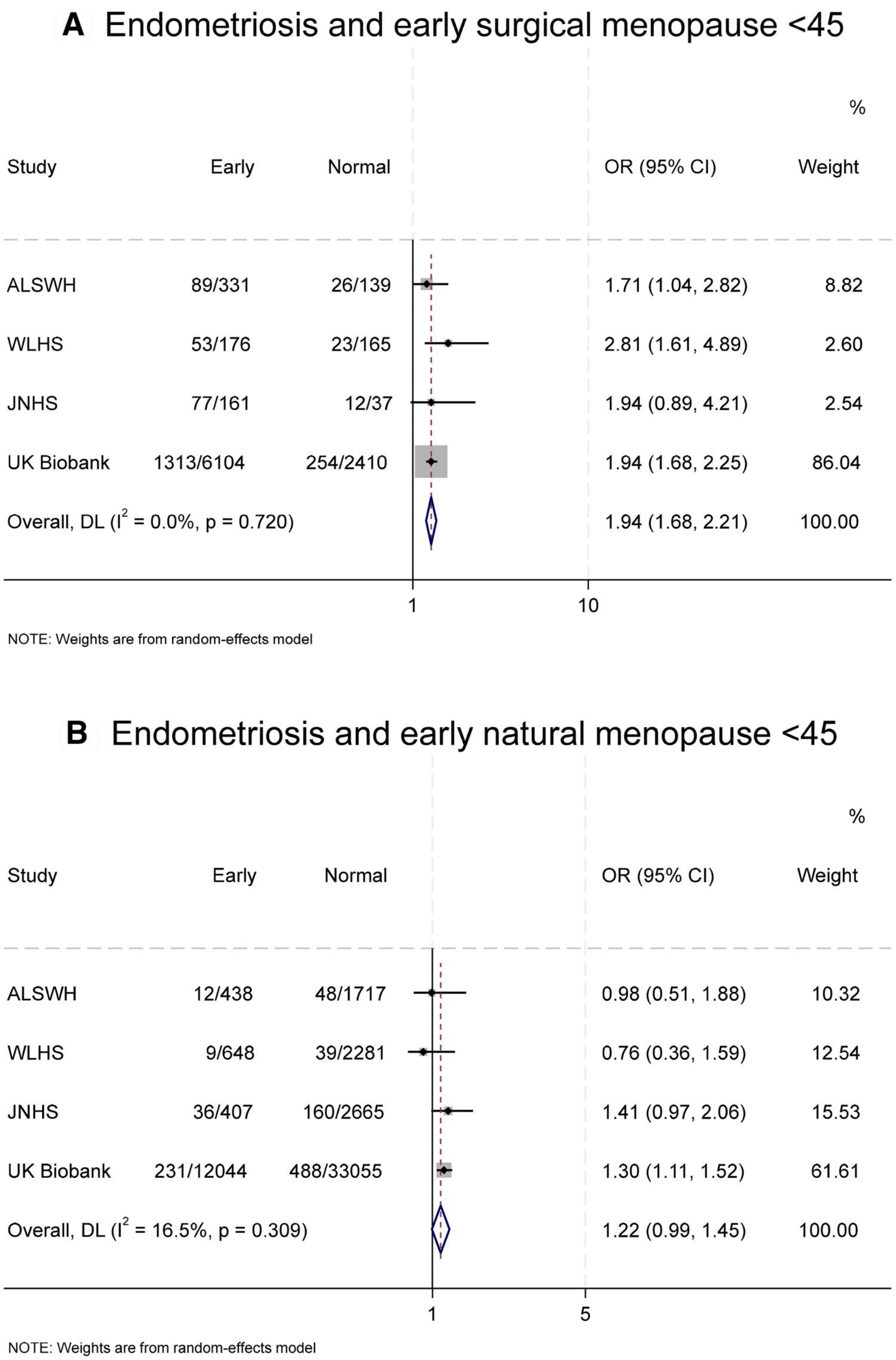

Supplementary Figure S2. Random-effect meta-analysis of the association between history of endometriosis and early menopause (A) early surgical menopause <45 years (reference: surgical menopause at age 50–51 years) and (B) early natural menopause <45 years (reference: natural menopausal at age 50–51 years). Data from NSHD were not included because of the small cell size of <5. Multinomial (polytomous) logistic regression models were used, and odds ratios (ORs) were fully adjusted for birth year, education level, race, smoking status, BMI, and age at menarche. DerSimonian–Laird (DL) method was used to estimate the heterogeneity variance (I^2 and *P*-values).
