# Supplementary material for: Association between endometriosis and type and age of menopause: a pooled analysis of 279 948 women from five cohort studies
Source: Hum Reprod. 2025 Apr 30;40(6):1210–9. doi: 10.1093/humrep/deaf068 (PMC12127511; doi:10.1093/humrep/deaf068)
Supplement: deaf068_Supplementary_Table_S1 [file deaf068_supplementary_table_s1.pdf]

OXFORD

human reproduction

Human Reproduction, 2025, 1–1
https://doi.org/10.1093/humrep/deaf068
Supplementary Data

Supplementary Table S1. Baseline characteristics of excluded and included participants.

Baseline characteristics	Excluded (n = 23 724)	Included (n = 279 948)
Birth year		
Born before 1940	386 (1.6)	6254 (2.2)
Born 1940–1949	8800 (37.4)	104 576 (37.4)
Born 1950–1959	8455 (35.9)	99 158 (35.4)
Born 1960 or later	5922 (25.1)	69 960 (25.0)
Race		
White	17 626 (74.3)	229 961 (82.1)
Black	436 (1.8)	3625 (1.3)
Asian	5369 (22.6)	44 356 (15.8)
Mixed/other	293 (1.2)	2006 (0.7)
Education level		
No formal education	1712 (7.7)	6986 (2.5)
≤10 years	6608 (29.5)	103 418 (36.9)
11-12 years	9502 (42.5)	93 116 (33.3)
University degree	4555 (20.4)	76 428 (27.3)
Smoking status at baseline		
Never smoker	12 574 (57.2)	166 226 (59.4)
Former smoker	5914 (26.9)	81 910 (29.4)
Current smoker	3514 (16.0)	31 812 (11.4)
Body mass index at baseline[a]		
Underweight	635 (3.8)	6034 (2.2)
Normal weight	8682 (52.2)	128 990 (46.1)
Overweight	4633 (27.9)	92 266 (33.0)
Obese	2676 (16.1)	52 658 (18.8)
Age at menarche		
≤11 years	2983 (18.4)	53 468 (19.1)
12 years	3772 (23.2)	59 364 (21.2)
13 years	4160 (25.6)	70 988 (25.4)
14 years	3068 (18.9)	54 788 (19.6)
≥15 years	2255 (13.9)	41 340 (14.8)
Number of children		
0	4021 (19.6)	53 361 (19.1)
1	2735 (13.3)	37 111 (13.3)
2	8302 (40.4)	119 611 (42.7)
3	3891 (19.0)	52 547 (18.8)
≥4	1584 (7.7)	17 318 (6.2)
History of infertility		
No	21 485 (90.9)	265 199 (94.7)
Yes	2140 (9.1)	14 741 (5.3)

Data were presented as number (%).

[a] Standard BMI cut-offs <18.5, 18.5–24.9, 25–29.9, and ≥30 kg/m^2 were used to categorise underweight, normal weight, overweight, and obese, respectively, in White, Black, and Mixed/Other women, while lower cut-offs <18.5, 18.5–22.9, 23–27.4, and ≥27.5 kg/m^2 were used in Asian women (reference in the Materials and methods section).
