# Supplementary material for: Association between endometriosis and type and age of menopause: a pooled analysis of 279 948 women from five cohort studies
Source: Hum Reprod. 2025 Apr 30;40(6):1210–9. doi: 10.1093/humrep/deaf068 (PMC12127511; doi:10.1093/humrep/deaf068)
Supplement: deaf068_Supplementary_Table_S2 [file deaf068_supplementary_table_s2.pdf]

Human Reproduction, 2025, 1–1
https://doi.org/10.1093/humrep/deaf068
Supplementary Data

Supplementary Table S2. The associations of history of endometriosis with type and age of menopause, additionally adjusting for infertility and number of children.

Type and age of menopause	History of endometriosis		Crude model[a]	Model 1[a,b]	Model 2[a,c]	Model 3[a,d]
	Yes	No	Estimate (95% CI)	Estimate (95% CI)	Estimate (95% CI)	Estimate (95% CI)
Surgical menopause (n = 279 948)						
Yes	3479 (33.6)	18 567 (6.9)	7.37 (6.92, 7.86)	7.58 (6.87, 8.36)	7.54 (6.84, 8.32)	7.50 (6.79, 8.28)
No	6888 (66.4)	2 251 014 (93.1)	Reference	Reference	Reference	Reference
Natural menopause (n = 279 948)						
Yes	3029 (29.2)	159 927 (59.3)	0.37 (0.27, 0.52)	0.40 (0.32, 0.49)	0.40 (0.33, 0.49)	0.40 (0.32, 0.49)
No	7338 (70.8)	109 654 (40.7)	Reference	Reference	Reference	Reference
Age at surgical menopause (n=22,046)						
Continuous age	45.2 ± 6.7	47.6 ± 6.5	−2.27 (−2.46, −2.08)	−1.59 (−1.78, −1.41)	−1.59 (−1.77, −1.42)	−1.52 (−1.66, −1.37)
Categorical age						
<40 years	697 (20.0)	2186 (11.8)	2.41 (2.19, 2.64)	2.10 (2.02, 2.19)	2.11 (2.02, 2.20)	2.03 (1.95, 2.12)
40–44 years	839 (24.1)	3073 (16.6)	2.12 (1.96, 2.29)	1.82 (1.73, 1.92)	1.82 (1.73, 1.92)	1.77 (1.68, 1.87)
45–49 years	993 (28.5)	5745 (30.9)	1.32 (1.13, 1.54)	1.21 (1.06, 1.38)	1.21 (1.06, 1.38)	1.20 (1.04, 1.38)
50–51 years	320 (9.2)	2450 (13.2)	Reference	Reference	Reference	Reference
52–54 years	320 (9.2)	2267 (12.2)	1.08 (1.03, 1.14)	1.10 (1.05, 1.15)	1.10 (1.05, 1.15)	1.10 (1.05, 1.15)
≥55 years	310 (8.9)	2846 (15.3)	0.84 (0.73, 0.96)	0.98 (0.82, 1.16)	0.98 (0.82, 1.16)	0.97 (0.82, 1.15)
Age at natural menopause (n = 162 956)						
Continuous age	49.9 ± 4.3	50.5 ± 4.3	−0.52 (−0.64, −0.40)	−0.38 (−0.46, −0.29)	−0.37 (−0.46, −0.28)	−0.27 (−0.35, −0.19)
Categorical age						
<40 years	60 (2.0)	2512 (1.6)	1.41 (1.20, 1.65)	1.37 (1.17, 1.60)	1.36 (1.17, 1.59)	1.23 (1.11, 1.36)
40–44 years	229 (7.6)	10 768 (6.7)	1.25 (1.14, 1.37)	1.25 (1.13, 1.38)	1.25 (1.13, 1.38)	1.18 (1.07, 1.30)
45–49 years	844 (27.9)	37 337 (23.3)	1.19 (1.12, 1.27)	1.16 (1.09, 1.22)	1.15 (1.09, 1.22)	1.12 (1.06, 1.19)
50–51 years	740 (24.3)	38 880 (24.3)	Reference	Reference	Reference	Reference
52–54 years	768 (25.4)	43 341 (27.1)	0.95 (0.89, 1.02)	0.96 (0.91, 1.02)	0.96 (0.91, 1.02)	0.98 (0.92, 1.04)
≥55 years	388 (12.8)	27 089 (16.9)	0.86 (0.77, 0.91)	0.96 (0.87, 1.06)	0.96 (0.87, 1.06)	0.97 (0.89, 1.06)

Data were presented as number (%), mean ± SD, hazard ratio (HR) and 95% CI, β and 95% CI, or odds ratio (OR) and 95% CI.

[a] Fine–Gray subdistribution hazards models were used to examine the type of menopause and account for a competing risk. When surgical menopause was the event of interest, natural menopause was treated as a competing risk. In contrast, when natural menopause was the event of interest, surgical menopause was treated as a competing risk. Generalised estimating equation (GEE) models were used to examine continuous and categorical age at menopause and account for correlated data. Study variability and within-study correlation were accounted by including an indicator for study as a covariate and indicating study as a cluster (crude model).

[b] Model 1 was adjusted for study, birth year, education level, race, smoking status, and BMI at baseline (in the hazards models, birth year, and education level were included as stratum variables).

[c] Model 2 was adjusted for covariates in Model 1 and age at menarche.

[d] Model 3 was adjusted for covariates in Model 2 and infertility and number of children.
