# Supplementary material for: Association between endometriosis and type and age of menopause: a pooled analysis of 279 948 women from five cohort studies
Source: Hum Reprod. 2025 Apr 30;40(6):1210–9. doi: 10.1093/humrep/deaf068 (PMC12127511; doi:10.1093/humrep/deaf068)
Supplement: deaf068_Supplementary_Table_S3 [file deaf068_supplementary_table_s3.pdf]

OXFORD
human reproduction
Human Reproduction, 2025, 1–1
https://doi.org/10.1093/humrep/deaf068
Supplementary Data

Supplementary Table S3. The associations of history of endometriosis with type and age of menopause, excluding studies with only self-reported data.

Type and age of menopause	History of endometriosis Yes	History of endometriosis No	Crude model[a] Estimate (95% CI)	Model 1[a,b] Estimate (95% CI)	Model 2[a,c] Estimate (95% CI)
Surgical menopause (n = 239 611)					
Yes	3313 (41.7)	18 282 (7.9)	7.32 (6.87, 7.81)	7.52 (6.81, 8.31)	7.49 (6.78, 8.27)
No	4641 (58.3)	213 375 (92.1)	Reference	Reference	Reference
Natural menopause (n = 239 611)					
Yes	2428 (30.5)	151 319 (65.3)	0.33 (0.31, 0.35)	0.35 (0.31, 0.41)	0.35 (0.31, 0.41)
No	5526 (69.5)	80 338 (34.7)	Reference	Reference	Reference
Age at surgical menopause (n = 21 595)					
Continuous age	45.3 ± 6.7	47.6 ± 6.5	−2.29 (−2.48, −2.10)	−1.59 (−1.78, −1.41)	−1.59 (−1.77, −1.41)
Categorical age					
<40 years	651 (19.6)	2129 (11.6)	2.45 (2.29, 2.63)	2.13 (2.06, 2.22)	2.14 (2.06, 2.22)
40–44 years	804 (24.3)	3027 (16.6)	2.15 (2.02, 2.29)	1.83 (1.74, 1.93)	1.84 (1.75, 1.93)
45–49 years	938 (28.3)	5656 (30.9)	1.33 (1.13, 1.55)	1.21 (1.06, 1.39)	1.21 (1.06, 1.39)
50–51 years	303 (9.1)	2411 (13.2)	Reference	Reference	Reference
52–54 years	310 (9.4)	2235 (12.2)	1.10 (1.05, 1.16)	1.12 (1.06, 1.18)	1.12 (1.07, 1.18)
≥55 years	307 (9.3)	2824 (15.4)	0.86 (0.76, 0.97)	1.00 (0.86, 1.16)	1.00 (0.86, 1.16)
Age at natural menopause (n = 153 747)					
Continuous age	50.0 ± 4.5	50.5 ± 4.3	−0.53 (−0.67, −0.39)	−0.39 (−0.48, −0.29)	−0.38 (−0.48, −0.28)
Categorical age					
<40 years	50 (2.1)	2437 (1.6)	1.30 (1.23, 1.38)	1.27 (1.18, 1.37)	1.27 (1.18, 1.36)
40–44 years	202 (8.3)	10 441 (6.9)	1.22 (1.08, 1.39)	1.23 (1.08, 1.40)	1.22 (1.07, 1.39)
45–49 years	636 (26.2)	34 808 (23.0)	1.16 (1.11, 1.20)	1.12 (1.08, 1.16)	1.12 (1.08, 1.16)
50–51 years	576 (23.7)	36 221 (23.9)	Reference	Reference	Reference
52–54 years	609 (25.1)	40 786 (27.0)	0.94 (0.85, 1.03)	0.94 (0.87, 1.02)	0.94 (0.87, 1.02)
≥55 years	355 (14.6)	26 626 (17.6)	0.83 (0.76, 0.90)	0.92 (0.87, 0.98)	0.92 (0.87, 0.98)

Excluding data from JNHS and NCDS.
Data were presented as number (%), mean ± SD, hazard ratio (HR) and 95% CI, β and 95% CI, or odds ratio (OR) and 95% CI.

[a] Fine–Gray subdistribution hazards models were used to examine the type of menopause and account for a competing risk. When surgical menopause was the event of interest, natural menopause was treated as a competing risk. In contrast, when natural menopause was the event of interest, surgical menopause was treated as a competing risk. Generalized estimating equation (GEE) models were used to examine continuous and categorical age at menopause and account for correlated data. Study variability and within-study correlation were accounted by including an indicator for study as a covariate and indicating study as a cluster (crude model).

[b] Model 1 was adjusted for study, birth year, education level, race, smoking status, and BMI at baseline (in the hazards models, birth year, and education level were included as stratum variables).

[c] Model 2 was adjusted for covariates in Model 1 and age at menarche.

© The Author(s) 2025. Published by Oxford University Press on behalf of European Society of Human Reproduction and Embryology.
This is an Open Access article distributed under the terms of the Creative Commons Attribution License (https://creativecommons.org/licenses/by/4.0/), which permits unrestricted reuse, distribution, and reproduction in any medium, provided the original work is properly cited.
Human Reproduction, 2025, 00, 1–10
https://doi.org/10.1093/humrep/deaf068
Original Article
